# Rare Bilateral Adrenal Haemorrhage with Addisonian Crisis: When Risk Factors Come in Droves

**DOI:** 10.1155/2020/8886537

**Published:** 2020-08-03

**Authors:** Anders Boisen Jensen, Elise Durand, Vojtech Pavlicek

**Affiliations:** ^1^Department of Endocrinology, Kantonsspital Münsterlingen, Spitalcampus 1, 8596 Münsterlingen, Switzerland; ^2^Department of Radiology, Kantonsspital Münsterlingen, Spitalcampus 1, 8596 Münsterlingen, Switzerland

## Abstract

Addisonian crisis is the life-threatening acute manifestation of adrenal insufficiency due to absolute or relative glucocorticoid deficiency. Adrenal haemorrhage is a rare condition of unknown incidence with the risk of adrenal insufficiency and death, not uncommonly first being diagnosed on the pathologists table. We report the case of a 68-year-old female patient with respiratory tract infection suffering acute life-threatening adrenal insufficiency caused by bilateral adrenal haemorrhages following orthopedic surgery while taking anticoagulation therapy. The patient rapidly deteriorated with hypotension, showing how important it is to consider a possible Addisonian crisis when this scenario occurs, especially with precipitating factors such as anticoagulant therapy, sepsis, or surgery.

## 1. Introduction

Addisonian crisis is the life-threatening acute manifestation of adrenal insufficiency due to absolute or relative glucocorticoid deficiency. Adrenal insufficiency based on pathophysiology can be classified as primary, secondary, or tertiary, where the latter two, also called “central adrenal insufficiency,” are caused by insufficient secretion of adrenocorticotropic hormone (ACTH) from the pituitary gland and corticotropin-releasing hormone (CRH) from the hypothalamus, respectively. Primary adrenal insufficiency (Addison's disease) results from destruction of the adrenal cortex and becomes manifest when at least 90% of the adrenal cortical tissue has been destroyed. In the developed countries, autoimmune disease accounts for approximately 80–90% of the cases, with metastatic disease, adrenal haemorrhage or infarction, drugs, and infections accounting for the rest. The yearly incidence in Europe is about 4.4–6.0 new cases per million with a prevalence of 93–144 cases per million [1].

Adrenal haemorrhage is rare with an unknown incidence but with the serious risk of adrenal insufficiency and death, not uncommonly first being diagnosed when an autopsy has taken place due to the nonspecific nature of the symptoms [2]. The diagnosis may be suspected or confirmed in a computed tomography (CT) or magnetic resonance imaging (MRI), demonstrating bilateral adrenal haemorrhage in about 20% of the total cases. The complete and potentially fatal loss of adrenal function after bilateral adrenal bleeding is practically given in every case. Risk factors for adrenal bleeding are advanced age, major surgery (especially cardiovascular and orthopedic surgery), infections (e.g., meningococcemia “Waterhouse-Friderichsen syndrome”), blunt abdominal trauma, significant hypotension, antiphospholipid syndrome, heparin-associated thrombocytopenia (HIT), anticoagulant use, and catecholamine excess (e.g., phaeochromocytoma) [3]. Kovacs et al. reported that, according to a case-control study with 23 patients with massive bilateral adrenal haemorrhage and 92 control patients, the variables with the strongest association with adrenal bleeding were hospital duration, thrombocytopenia, heparin use, sepsis, and hypotension [3]. The incidence of adrenal bleeding on anticoagulation therapy is rare and only a third of the patients with adrenal haemorrhage are anticoagulated [4].

We report the rare case of a patient with acute adrenal insufficiency caused by bilateral adrenal haemorrhage whilst on anticoagulation therapy following orthopedic surgery and a respiratory tract infection, demonstrating how the early diagnose and treatment of an Addisonian crisis can be lifesaving.

## 2. Case Presentation

A 68-year-old female was admitted to the emergency room from an orthopedic rehabilitation clinic with tiredness, vertigo, nausea, vomiting, and bilateral flank pain for the last three days. Eleven days earlier, she underwent a total knee prosthesis surgery on the left side. Before admission, her blood pressure values from the rehabilitation clinic showed no hypotensive episodes. Due to an atrial fibrillation, the patient was being anticoagulated with phenprocoumon. The phenprocoumon was stopped days prior to her surgery, during which she was anticoagulated with unfractioned heparin, and then restarted a few days postoperatively. There was no record of her suffering any blunt trauma or receiving corticosteroid therapy. Three days before admission, an abdominal ultrasound scan showed no abnormalities in the perirenal regions. Due to an aspiration pneumonia, piperacillin/tazobactam antibiotics had been started three days prior to admission. Clinically the patient was lethargic and apyrexial, her blood pressure hypotensive with 84/57 mm Hg, and her heart rate arrhythmic tachycardic with 138 BPM. No cutaneous or mucosal hyperpigmentation was evident. Her left knee was painless to palpation, slightly warm with swelling but no redness.

**Figure 1:**
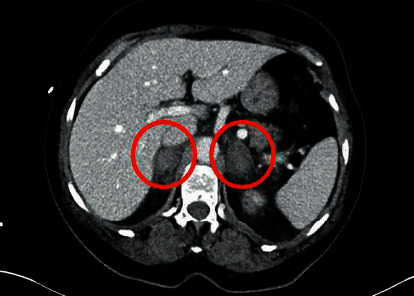
CT of the abdomen at admission (horizontal plane).

The key laboratory findings at admission were hypocortisolism with hypokalemia and hyponatraemia, further findings are listed in Table 1. Usually hypocortisolism causes hyperkaliemia, but our patient was at admission suffering from vomiting which speaks for the hypokaliemia being caused by gastrointestinal loss of potassium; the antibiotics of the patient piperacillin/tazobactam can however also cause this electrolyte disturbance. ACTH was unfortunately not measured until four days later at which time it was still elevated at 173 ng/l, consistent with a non-ACTH dependent adrenal aetiology of the hypocortisolism. Urine culture and serial blood cultures (all taken whilst on antibiotic therapy) were clear. Chest and left knee radiography were normal. A CT of her abdomen showed bilateral hyperdense oval enlargement of the adrenal glands (right 27 × 16 mm, left 32 × 21 mm) consistent with acute adrenal haemorrhages, no sign of malignant tumor was demonstrated (Figure 1).

Later the patient developed a severe anaemia (hemoglobin 70 g/l) without any clinical signs of blood loss, a gastroscopy revealed no signs of bleeding. The adrenal haemorrhages were, due to their moderate size on the CT, felt to not be the cause of her reduced hemoglobin why we interpreted the culprit to be a fluid shift in the context of the Addisonian crisis. The hemoglobin improved significantly to a value of 92 g/l at discharge without a blood transfusion. Before discharge, a HIT with positive platelet factor 4 antibodies was diagnosed due to falling thrombocyte count whilst on prophylactic unfractioned heparin, and heparin was replaced with fondaparinux and by discharge changed to rivaroxaban, when a therapeutic anticoagulation was deemed safe again after the adrenal haemorrhage. The 21-hydroxylase, antiadrenal antibody, and antiphospholipid syndrome antibody essays were negative, making an autoimmune cause of the primary adrenal insufficiency unlikely. An ACTH stimulation test was not performed given the clinical presentation, very low serum-cortisol, and CT-findings, leaving little doubt about the presence of an adrenal crisis and its aetiology.

With the clinical constellation at admission of acute flank pain, hypotension, hyponatremia, and radiological signs of an adrenal bleeding whilst on anticoagulation, an Addisonian crisis as the acute manifestation of primary adrenal insufficiency was strongly suspected. Intravenous hydrocortisone therapy and a saline infusion were immediately started, and phenprocoumon was stopped. The patient was referred to the intensive care unit (ICU) for circulatory support with norepinephrine and further intravenous fluids until her condition stabilised 4–6 hours later. The hydrocortisone was tapered down over the next 4-5 days until she was on a split dose of 30 mg/day per orally, and subsequently fludrocortisone 0.1 mg/day was initiated.

**Figure 2:**
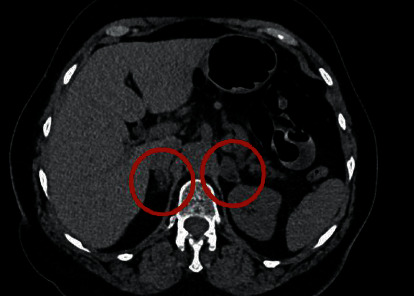
Follow up CT of the abdomen (horizontal plane).

On review as an outpatient, the condition of the patient was back to baseline. A follow-up unenhanced CT (Figure 2) was performed 4 months later demonstrating no signs of malignant tumor and regression of the enlarged adrenal glands (right 22 × 12 mm, left 20 × 12 mm), a sign that the haematoma was slowly resolving [5]. It is important that patients should have follow-up scanning until resolution of the haematoma and that the possibility of underlying pathological processes such as malignancy is considered, especially in patients with unilateral adrenal haemorrhages.

After starting fludrocortisone 0.1 mg a day, the sodium level was 141 mmol/l, the potassium level 4.6 mmol/l (both in the normal range) and the renin level in the lower normal range with 4.8 m*U*/l, which could make one suspect that the patient may not have had aldosterone deficiency despite glucocorticoid deficiency or that the patient on her own had ingested more hydrocortisone than was prescribed, which through the mineralocorticoid effect of hydrocortisone would have lowered the renin level.

The continuous adjustment of the medication takes place in the outpatient setting. Five months after the first diagnosis of primary adrenal insufficiency, despite on several occasions being given verbal and written information about adrenal insufficiency and its medical management including an emergency card, the patient was again admitted to the intensive care unit with an Addisonian crisis due to an influenza-infection with fever up to 40°C having failed to increase her hydrocortisone dose. She survived after receiving high-dose intravenous hydrocortisone and fluid resuscitation in the ICU and was discharged after being given repeated information about sick day rules when experiencing stressful situations.

## 3. Discussion

The anatomy of the adrenal vasculature may play an important role in the development of adrenal haemorrhage. The adrenal glands are one of the best vascularised organs in the body; the adrenal vasculature is supplied through three arteries going into the adrenal tissues but at the same time having a limited venous drainage through only one adrenal vein. The arteries penetrate the surface of the adrenal gland; divide into 50–60 branches that form a vascular plexus around the zona reticularis. This rather abrupt arteriovenous transition zone constitutes a so called “vascular dam,” with a propensity to cause congestion of the vasculature owing to the anatomical “bottleneck” when the blood is further drained into medullary sinusoids and ultimately into the single adrenal vein [3]. According to Fox, the eccentric longitudinal smooth muscle fibres of the adrenal vein could, in situation of vascular contraction, cause local stasis and pockets of turbulence, in which venous thrombosis would form, causing vascular wall necrosis leading to a secondary haemorrhagic event [6]. Dobbie and Symington suggested that this mechanism for build-up of venous stasis actually has a physiological role, allowing the adrenal cortex to become engorged with and, using the elastic fibres in the venous wall, subsequently explosively expel a large volume of blood containing adrenal cortical hormones out of the adrenal gland into the systemic circulation, allowing for an acute hormonal effect [7]. An additional thrombogenic factor in stressful situations is the ACTH and catecholamine secretion which increases the arterial blood flow to the adrenal glands, causing adrenal venous stasis and inducing platelet aggregation, both of which increase the risk of an infarction and secondary haemorrhage [2].

In our patient, several risk factors for an adrenal haemorrhage were identified: anticoagulation therapy, heparin induced thrombocytopenia, recent surgery, and infection. Most of the reported adrenal haemorrhage cases whilst on anticoagulant therapy occurred within the first 2–4 weeks after initiation [8, 9]. Our patient, however, had been using phenprocoumon for several years. In a review by Rao, after surgery, adrenal bleeding is most commonly seen in the first fifteen days postoperatively [10], which was also the case in our patient. She had no vasodilatory therapy, which could have had a protective effect against the development of adrenal haemorrhage by increasing the perfusion in ischemic vessels [3]. The administration of heparin before and after the knee surgery, in this patient with slightly decreased platelet count of 135 G/l on the day of admission, could have played a significant role in the development of the bilateral adrenal bleedings, since Kovacs defined heparin for a minimum of 4 days and thrombocytopaenia as high risk factors for adrenal haemorrhage [3]. Her platelet count on the day before admission was however normal at 173 G/l, which in our view excludes the weeks later established diagnosis of a HIT as precipitating factor of the adrenal bleeding.

In our opinion, the reason for the adrenal bleeding in this patient is most likely the stress induced by the surgery and the infection on top of the preexisting anticoagulation therapy. The anticoagulation therapy had been used for years and by admission, her INR-values were in the therapeutic range, which gives support to the notion of her infection and surgery as the main triggers for the adrenal bleeding.

Rapid clinical deterioration in hypotensive patient with hyponatremia should always lead to investigations for a possible Addisonian crisis, and the prompt intravenous administration of fluids and hydrocortisone can be life-saving and should be given while awaiting diagnostic laboratory test results. An Addisonian crisis due to adrenal haemorrhage should be suspected in patients showing signs such as abdominal/flank/back pain, signs of occult haemorrhage, and adrenal insufficiency, especially in the presence of coinciding risk factors such as anticoagulation therapy, recent surgery, and infection. A CT of the adrenal glands can help to establish the diagnosis.

## Figures and Tables

**Table 1:** Laboratory studies at admission.

Parameter	Result (CH units)	Normal range
Sodium	124 mmol/l	136–145 mmol/l
Potassium	2.6 mmol/l	3.4–5.0 mmol/l
Serum osmolality	258 mmol/kg	280–300 mmol/kg
Blood urea nitrogen	3.0 mmol/l	<11.9 mmol/l
Chloride	87 mmol/l	96–108 mmol/l
pH	7.40	7.37–7.45
Bicarbonate	27 mmol/l	21–26 mmol/l
Glucose	5.4 mmol/l	3.9–6.4 mmol/l
Creatinine	47 *µ*mol/l	44–80 *µ*mol/l
eGFR (CKD-EPI)	98 ml/min	>90 ml/min
Random serum-cortisol	39 nmol/l	80–690 nmol/l
ACTH (day 4)	173 ng/l	<61 ng/l
TSH	2.23 mU/l	0.27–4.2 mU/l
C-reactive protein	158 mg/l	<5 mg/l
Hemoglobin	118 g/l	120–160 g/l
Eosinophils	0.1 G/l (0.7%)	<0.7 G/l (2–4%)
Thrombocytes	135 G/l	150–375 G/l
INR (international normalized ratio)	2.8	2.0–3.0
Antiadrenal & 21-hydroxylase antibodies	Negative	
Antiphospholipid syndrome antibodies	Negative	
Platelet factor 4 IgG antibodies	24.6 U/ml	<1.0 U/ml
Urine culture	Negative	
Blood culture	Negative	

## Data Availability

Request should be sent to andersboisen.jensen@kssg.ch for further data.
